# Supplementary material for: Kernel-DMD for multiome data integration and control
Source: PLoS Comput Biol. 2026 Mar 31;22(3):e1014029. doi: 10.1371/journal.pcbi.1014029 (PMC13038008; doi:10.1371/journal.pcbi.1014029)
Supplement: S1 Appendix — Specifically, the analogy between the sigmoidal kernel and Hill function dynamics is made clearer along with the derivation of the sensitivity gain matrix (29). (DOCX) [file pcbi.1014029.s004.docx]

S1 Appendix: Supplementary information

**Design of ‘Hill-like’ kernel with a-priori pathway constraints**

In more detail, Hill functions transition from a value of 0 to 1 in a sigmoidal shape, after a threshold is exceeded. This is a useful representation of the on or off transcription of a gene according to the concentration of a transcription factor after exceeding a certain threshold. The sigmoid kernel can approximate Hill function dynamics due to similarity to sigmoidal dynamics $g\left( x \right)=\frac{1}{1+ⅇ^{-\alpha(x-c)}}$, under a proper choice of hyperparameters. As shown in (36) the main difference is power laws versus exponentials in the denominator. Contrary to Hill functions, sigmoidal functions can be derived as gradients of free energy functions (see (36) for the derivation) that represent the solution of equations of motion within an energy landscape. As shown in (36), the rate of change equation of each chemical species $x_{i}$ is derived as

$\frac{ⅆv_{i}(t)}{ⅆt}=g\left( -\frac{1}{2T}\left( -\sum_{j} \gamma_{ji}x_{j}\left( t \right)- c_{i} \right) \right)- {\beta x}_{i}\left( t \right)$ (1)

where the first part is the sigmoidal approximation of Hill interaction dynamics, $g\left( x \right)=\frac{1}{1+ⅇ^{-x}}$, *T* is a noise term, *γ* the interaction strength between molecules *j* and *i*, *c* a forcing term and – *βx_i_(t)* a linear degradation term with rate constant *β*.

We kernelized this as shown in Table 1, with *γ* as a coefficient of the inner product representing the interaction strength between features and *c* as the forcing term. We ignored *T* and assumed its effect through *γ* and *c*. The linear degradation term was represented as a linear kernel of the input feature space. Each term of (1) had its own weight (*c_s_* for the sigmoid and *c_l_* for the linear part). We made sure the value *γ* was small enough (1e-3) to control the scale of similarity in the dot product of the inputs. Larger *γ* values lead to more curvature in the sigmoid and steeper decision boundaries, whereas smaller values lead to a more generalised decision boundary that also avoids vanishing gradients and reduces the effect of individual points that might contribute to noise. The bias term

$c= -\gamma+\frac{1}{n^{2}}\Sigma_{i=1}^{n}\Sigma_{j=1}^{n}{(XX^{T})}_{ij}$ (2)

shifts the kernel function along the output axis and ensures that the decision boundary of the sigmoid falls well within the base state of the data and follows the curvature set by *γ*. The right choice of *γ* and *l* also ensure that the Jacobian linearization of the kernelized dynamics does not have vanishing parts as the base state chosen falls well within the decision boundary of the sigmoid kernel.

**Sensitivity gain matrix identifies glycolytic enzymes as points of control**

If the linear state-space system is expressed in continuous time as

$\frac{d}{dt}x(t)=Ax\left( t \right)+Bu\left( t \right)$ (3)

substituting the control law

$u\left( t \right)= -Kx\left( t \right)$ (4)

gives the closed-loop system

$\frac{d}{dt}x(t)=\left( A-BK \right)x(t)$ (5)

Considering a perturbation of both state and input

$x\left( t \right)= x_{0}\left( t \right)+\Delta x\left( t \right), u\left( t \right)= u_{0}\left( t \right)+\Delta u\left( t \right)$ (6)

the perturbed system becomes

$\frac{d}{dt}x_{0}\left( t \right)+\Delta x\left( t \right)=\left( A-BK \right)\left( \frac{d}{dt}x_{0}\left( t \right)+\Delta x\left( t \right) \right)+B(u_{0}\left( t \right)+\Delta u\left( t \right))$ (7)

At steady state $x_{0}\left( t \right)=0$ so,

$\frac{d}{dt}\Delta x\left( t \right)=\left( A-BK \right)\Delta x\left( t \right)+B\Delta u\left( t \right)$ (8)

And if at steady state $\frac{d}{dt}\Delta x\left( t \right)=0$, then

$\Delta x\left( t \right)={(\tilde{A_{1}}-BK)}^{-1}B\Delta u\left( t \right)$ (9)

and the effect of the rate of change in *u* on the rate of change in *x* is given by the gain matrix *S*.
