# Supplementary material for: Kernel-DMD for multiome data integration and control
Source: PLoS Comput Biol. 2026 Mar 31;22(3):e1014029. doi: 10.1371/journal.pcbi.1014029 (PMC13038008; doi:10.1371/journal.pcbi.1014029)
Supplement: Attachment — Submitted filename: Reviewers_reply.docx [file pcbi.1014029.s005.docx]

***We thank the editors and reviewers for their valuable insights and suggestions. Below we give our reply to the reviewers’ comments.***

**Reviewers' comments:**

Reviewer's Responses to Questions

**Comments to the Authors:
Please note here if the review is uploaded as an attachment.**

Reviewer #1: The manuscript uses a dynamical-systems approach (kernel-DMD) to integrate multi-omics time-series data in two Clusia species, revealing causal network drivers of C3 versus CAM photosynthesis and identifying biomarkers and control strategies relevant for crop bioengineering.
The authors have done substantial work in the area of multiome network integration. However, my primary concerns are as follows:
1. The authors should briefly introduce the specific strategy used, i.e., the components of the proposed kernel-DMD framework, rather than only emphasizing its significance. The significance may be included, but it should relate to the contribution and importance of kernel-DMD itself.

*ANSWER: First of all, we thank the reviewer for very helpful comments on our manuscript. Specifically, we follow these suggestions and introduce DMD, kernel-DMD and its kernel component with more emphasis in the introduction along with more references on its applications and what it contributes to the analysis. More technical details are included in the methods*.

2. The Introduction and Methods sections are currently merged, which results in high cognitive load when reading. I strongly recommend separating them into two independent sections. Additionally, when presenting the Methods, a top-down structure should be used, starting with an overview followed by detailed subsections.

*ASNWER: We thank the reviewer very much for the useful suggestions. We completely restructured the manuscript accordingly. We have now clearly separated the Introduction and the Methods section and generated a fully new figure 1 to introduce all technical steps of our study step-by-step. Technical parts that were in the Introduction were moved to the Methods section (specifically the DMD explanation part). The methods section was extended by adding technical details that were in the results section, in a hierarchical manner starting from a general overview of the workflow and then building up in subsections. We specifically moved the Methods section before the Results and Discussion because we believe the technical workflow of the manuscript (as also shown in figure 1) needs to proceed the results for a thorough understanding of how the results were generated. The main output of the manuscript is part of how methods were implemented and the whole pipeline is what we want to highlight with results indicating the application of the methods.*

3. Why is the sigmoid kernel more suitable than Gaussian or polynomial kernels for multi-omics dynamic modeling? Is there any risk of overfitting? I recommend adding experimental comparison rather than relying solely on theoretical derivations to support this choice.

*ANSWER: 🡪 We compared the accuracy between different kernels based on average data reconstruction accuracy (R^2^ score) over 100 iterations and their standard deviations (Table 2 in the manuscript). Upon careful evaluation of the kernels following Reviewer’s comment no. 3, the success of the sigmoid kernel in terms of R^2^ score over the gaussian and polynomial was found to be attributed to its second linear part which is also why the linear kernel results in high accuracies as well. Experimental comparison of the qualitative results of each kernel based on sensitivity values in the last section of the paper, concluded that the sigmoidal kernel (only when combined with its linear part) gave results more aligned with the literature on what is known about the differences between CAM and C3. In particular it separated the features related to glycolysis that lead to PEP production (PFK3, GAPN, FBA2 and SPS1) and other strong CAM related features (PPC16, MDH1) as features with positive feedback in contrast to features related to photorespiration (RBCS, GDCSP, GDCSH, GDCST) as features with negative feedback after applying control, in agreement with the differences uncovered by eigendecomposition comparisons. This clear distinction was not made by other kernels without the linear part, so we concluded that the tailor-made sigmoidal kernel can produce results in alignment with what is known in terms of biology in this case. We noted this in the manuscript. In addition, without the linear part, no kernel could phenocopy the outputs in the control pipeline.*

*ANSWER: 🡪 We ensured there is no risk of overfitting by carrying out a leave-3-out cross validation 10 times, which resulted in high accuracies (0.7 for both data and outputs). We also note this in the manuscript as before.*

4. Equation (13) involves multiple objectives, but the manuscript does not sufficiently explain how the weights are determined or how they influence the optimization outcomes.

*ANSWER: 🡪We added more details on how the weights are optimized and their significance in the mutiobjective function. We also noted their feasibility as a direct outcome of the representation theorem in kernel learning.*

5. Using HSV to rank controllability and observability is meaningful, but questions remain regarding the stability of HSV. Does the feature list converge across iterations? A more thorough explanation or sensitivity analysis would strengthen this part.

*ANSWER: 🡪We added figure 5A to show how many times a feature was retained after data reduction over 100 iterations. This clearly indicates the robustness of HSV selection as it shows the top features that were selected in every iteration by appending each feature that was selected after data reduction using HSV values in each iteration and then counting them over 100 iterations (Frequency shown in the y-axis of the figure for C. rosea and C.major).*

6. The keywords need to be consistent throughout the entire manuscript. For example, but not limited to, “multi-ome network integration” and “multiome network integration.” Please check the wording throughout the manuscript and revise accordingly.

*ANSWER: 🡪All keywords related to “multiome” were made consistent.*

7. Although the authors provide a Zenodo repository for data and code availability, the files appear to be marked as Restricted. A publicly accessible GitHub repository is recommended for reproducibility.

*ANSWER: 🡪The code and data are now available in a publicly accessible Github repository as well as Zenodo (data on Zenodo:* DOI: 10.5281/zenodo.17370335; code on Zenodo: DOI: 10.5281/zenodo.18314409)

8. Given the manuscript’s relevance to network science, the authors are encouraged to cite the following paper(s) to further strengthen the background and context.
DOI:10.1038/s41551-024-01312-5, DOI:10.1109/TSMC.2025.3578348, DOI:10.1109/TSMC.2025.3572738

*ANSWER: 🡪We cited the suggested papers in appropriate sections of the Introduction and Methods.*

Reviewer #2: This is a high quality, interesting and relatively novel study. I commend the authors on their work.

*ANSWER: 🡪We thank Reviewer 2 very much for their encouraging comments.*

Reviewer #3: Dear authors and editor,

I have carefully read the manuscript entitled “Kernel-DMD with control for phenocopying of complex biological systems,” in which you describe and apply kernel dynamical mode decomposition to uncover photosynthetic dynamics from multiome data in Clusia major and Clusia rosea. I find this work potentially valuable to the biology community. However, in its current form, and from the perspective of someone with a modeling and statistical analysis background (though not an expert in DMD methods), I found the manuscript rather difficult to follow. Additionally, the overall purpose of the paper is not clearly articulated. While you demonstrate that the analysis yields insights into the photosynthetic processes of Clusia, and I understand that you also intend to showcase the broader applicability of the method, the presentation is not sufficiently explanatory. As a result, the manuscript is not easily accessible to readers outside the immediate field. I also believe that the manuscript requires substantial polishing in its organization, flow, and figures.
Regarding the introduction, parts of it read smoothly, but others feel out of place (see specific comments below). More importantly, the introduction does not adequately prepare the reader for the analyses that follow, nor does it explain why certain methodological choices are made later in the paper. In the description of the approaches used in the results, it would be very helpful to provide clearer motivation and intuition for the analytical decisions you make and to explain why these steps are necessary. Furthermore, key methodological components are intermixed with more technical or secondary details. I would recommend moving some of the more involved technicalities to supplementary material or to designated subsections in the Methods, allowing the main text to focus on conceptual clarity.

*ANSWER: We thank the reviewer very much for these very useful comments. We followed the suggestions and completely restructured the manuscript accordingly.* *-> The introduction now includes a clearer motivation and short overview of the workflow as well as why each section in the methods was chosen and its relative importance for the results. We have prepared a completely new figure 1 summarizing the organization and the flow of the study.* *Technical details in the results section were moved to the methods with clearer intuition as to why they were chosen*. *We further revised the results section to provide a better conceptual flow and more focus on the outcomes of the study. The organization of the manuscript follows now a clear concept, the introduction as motivation for the study, the methods section for covering all the technical details and the results section separated in part 1 System Identification and part II System Control which is also reflected in figure 1.*

I also found the merged Results and Discussion section difficult to navigate. It becomes unclear where the presentation of results ends and where broader interpretation begins, particularly since the conclusion appears to begin around line 751 within a section titled “Sensitivity gain matrix identifies glycolytic enzymes as points of control.” It is fine to discuss results immediately after presenting them, but I would still suggest including a dedicated general discussion section to synthesize the main findings and their broader implications.

*ANSWER: 🡪 We separated Results from Discussion, clearly indicating raw results in the Results section (including optimization outcomes, choice of kernel, data, output and modal reconstruction accuracies, phenocopy prediction accuracies and eigenmode topology results, sensitivity results) with a more thorough discussion on sensitivity matrix results and how they compare to eigenmode topology results in the discussion section as well as more general implications of our method to the wider community.*

Several figures require improvement. Many are missing axis labels (e.g., Figures 3, 4, and 5 lack x-labels; Figure 8 lacks a color legend), and Figures 4 and 7 appear to be missing units for the decomposed dynamics of malic acid, H₂O, etc. Clearer figure presentation is essential for readability.

*ANSWER: We looked through all figures and corrected the mentioned mistakes. We also generated figure 1 completely new.*
*🡪We added x-labels and color legend where required, as well as z-score unit labels for the outputs where they are found.*

Specific comments:

Abstract: “We enhance the applicability of the Koopman operator to multiome integration.”
The meaning of enhance is unclear. Perhaps enable or demonstrate would be more precise.

*ANSWER🡪 We made the suggested change of the word “enhance” to demonstrate.*

Line 58: You may consider supplementing “vector that characterizes the state of the system” with intuition—for example, that could represent the abundance of molecular components.

*ANSWER: 🡪 We added a description of what the x vector represents in terms of multiome features.*

Line 81: “Data-driven approaches ((17), (18), (19)) have emerged…”
I find the rest of this paragraph distracting from the main point and the flow. I would suggest simplifying it and focusing directly on the conceptual gap your work addresses.

*ANSWER: -> We reduced the length of the paragraph and the emphasis on the differential Jacobian reconstruction is now within the context of previous data-driven tools and what is missing that can then be accounted for by DMD.*

Line 328: “Within the sigmoidal kernel we also implemented the graph Laplacian for integration of a priori knowledge of molecular pathways…”
It is not entirely clear how this implementation works. My assumption is that the graph Laplacian imposes constraints on allowable connectivity, but this should be clarified.

*ANSWER: 🡪We added a more thorough explanation of how the graph Laplacian influences the sigmoidal kernel.*

Line 350: The dash/apostrophe in the text differs from the symbol used in the corresponding equation.

*ANSWER: 🡪 We clarified the use of the dash that distinguishes the concatenated inputs that correspond to one time-step ahead.*

Line 345: “We map inputs to physiological outputs Y in the following multi-objective function…”
I understand the intention here, but it would be useful to elaborate somewhere on the reasoning behind this formulation. As far as I understand, you aim to map X to Y and then interpret the DMD results to identify what drives the phenotypic outputs. Clarifying that these outputs quantify phenocopies would also help.

*ANSWER: 🡪 We further elaborated on the link between learned Koopman dynamics and output measurements Y through eigenmode predictions of each output and also as part of the phenocopy of the CAM signature within a C3 molecular background.*

Line 364: “Linear operator from Taylor series expansion.”
This feels out of place. Please motivate why this expansion is introduced and how it relates to the main methodological narrative.

*ANSWER: 🡪 we clarified that the Taylor series expansion and the derivation of the linear Koopman operator through the kernel gradient is essential in extracting a meaningful representation of local dynamics of the multiome network, as also outlined in the original LANDO approach (1). For the phenocopying we rely on linear model predictive control; therefore, linearization is a prerequisite to the optimal control strategy later on.*

Closing remarks:
In summary, I believe the underlying work has value, and the methodology has the potential to be useful to the broader community. However, in its current form, the manuscript requires major rewriting to make it accessible, coherent, and reader-friendly. With substantial improvements to organization, clarity of exposition, motivation of methodological choices, and figure presentation, the paper could be significantly strengthened.

Reviewer #4: The study uses kernel-DMD to modeling photosynthesis. However, several issues regarding clarity, organization, notations, and biological interpretation need to be addressed before the manuscript can be considered for publication. My detailed comments are as follows:

1. The title uses the word "complex biological systems" is so big.

*ANSWER 🡪We changed the title to "Kernel-DMD for Multi-Omics Integration and Control"*

2. Update the references or revise the format, such as [16], [20].

*ANSWER 🡪 We updated the references.*

3. Please check the word "multiome" and "multi-omics"

*ANSWER: 🡪We made the word “multiome” consistent throughout the paper*.

4. Figure 1 requires revision for clarity and readability.

*ASNWER: 🡪 We changed figure 1 to contain more clear steps of both the System Identification and System Control parts that follows the flow of our methods section and also includes some indications of main results so that the workflow becomes clearer.*

5. Figure 2 illustrates photosynthesis pathways, which are not central to the methodological framework or the main results. I recommend moving this figure to the Supplementary Information.

*ANSWER: 🡪 We moved this figure to the Supplementary Information*

6. The main text is heavily interwoven with biological explanations and mathematical derivations. This mixture obscures the key contributions and makes it difficult for readers to follow the main arguments. I suggest restructuring the text so that the mathematical framework and biological interpretations are presented more clearly and separately.

*ANSWER: We thank the reviewer very much for these very useful comments. We followed the suggestions and completely restructured the manuscript accordingly. The introduction now includes a clearer motivation and short overview of the workflow as well as why each section in the methods was chosen and its relative importance for the results. We have prepared a completely new figure 1 summarizing the organization and the flow of the study. Technical details in the results section were moved to the methods with clearer intuition as to why they were chosen. We further revised the results section to provide a better conceptual flow and more focus on the outcomes of the study. The organization of the manuscript follows now a clear concept, the introduction as motivation for the study, the methods section for covering all the technical details and the results section separated in part 1 System Identification and part II System Control which is also reflected in figure 1.*

7. Line 146: In Eq. (2), please check and correct the notation for x_{t+1}

*ANSWER: 🡪We deleted the parenthesis around t+1.*

8. Line 170: The term “POD” is used without definition. Please provide a clear explanation upon first appearance.

*ANSWER: 🡪 We made the POD abbreviation clear to Principal Orthogonal Decomposition*

9. Line 259: In Eq. (8), should the symbol on the right-hand side be "X" as in Eq.(4)?

*ASNWER 🡪 Yes, we corrected it for consistency, as well as in equation 7.*

10. Line 266: Why is "alpha =3" chosen? Please discuss the rationale and whether alternative parameter choices affect the results.

*ANSWER🡪The choice of alpha=3 was arbitrary in order to avoid overfitting in the multi-objective function. When changing the value, output accuracies did not change. We did notice an improved reconstruction accuracy of the phenocopy of PPD when alpha=1 so we kept it at 1 and changed it in the manuscript.
Values below 0.01 lowered the phenocopy reconstruction accuracies of most outputs.*

11. Line 341: The subsection number appears incorrect (“3.1.3”?). Please revise.

*ANSWER 🡪We removed subsection numbering.*

12.Line 375: Is X_j treated as fixed or variable in this context? Please clarify.

*ANSWER 🡪We clarified that each j is a variable index corresponding to different omics data types.*

13. Line 381: The manuscript refers to "section 3.2," but this section is missing or mislabeled.
*ANSWER🡪 We deleted this reference.*

14. Line 398: "with 0 standard deviations" is that true? or just near 0?
*ANSWER🡪We changed that to s.d. <0.05*

15. The biological meaning of the different modes in Figures 3 and 4 is unclear. Please provide more explanation or biological context.

*ANSWER🡪 We thank the reviewer for this important point. Figures 3 and 4 indeed present the eigenvalues, eigenmode amplitudes, and mode-specific reconstruction accuracies from one representative kernel-DMD iteration. Because kernel-based system identification involves stochastic initialization and resampling, the precise shape of individual eigenmodes varies across iterations, and therefore Figures 3 and 4 are intended only to illustrate the spectral structure and reconstruction behavior of the method.*

*To address the reviewer’s request, we have clarified this explicitly in the revised Results section. We now explain that biological interpretation is not drawn from a single iteration, but rather from a consensus analysis across 100 iterations, where only eigenmodes that consistently predict CO₂ gas-exchange dynamics (R² > 0.3) are retained. This consensus approach identifies the stable spatial and temporal patterns of the system and forms the basis of our biological conclusions.*

*We have added to the Results section a concise description of how the consensus eigenmode topology (Fig. 4; Supplementary Tables) reveals biologically interpretable modules—such as glycolysis and the TCA cycle in C. rosea and photorespiration in C. major—and how these stable structures relate to differences between CAM and C3+CAM photosynthesis.*

16. Figure 5 presents eigenmode topology, but the interpretation is difficult for readers. Additional explanation or annotation is needed.
*ANSWER: 🡪 We agree that the biological interpretation of Figure 4 can be further clarified. In the revised manuscript, we have substantially improved both the visual annotation and textual explanation associated with Figure 4.*

*Specifically, we have:*

- *Added pathway-level annotations directly onto the eigenmode topology (e.g., glycolysis, TCA cycle, photorespiration), allowing readers to immediately see which biological modules are represented in each consensus eigenmode cluster.*
- *Highlighted key hub features (e.g., PEPC, PPCK1, AGPP, RBCS) with distinct colouring and labels to emphasize their functional relevance to CAM or C3 dynamics.*
- *Included a short explanatory caption extension describing how to interpret the topology—i.e., that edges represent high-frequency co-occurrence across eigenmodes over 100 iterations, and pathways correspond to dynamical modules influencing CO₂ gas exchange.*
- *Highlighted in the corresponding Results subsection a clear narrative of how the consensus topology reveals (i) enhanced glycolytic and organic-acid-associated fluxes in C. rosea, and (ii) enhanced photorespiratory/cofactor cycling activities in C. major. This connects the visual network to the functional biological differences between CAM and C3+CAM systems.*

*These modifications ensure that Figure 4 is not only visually clearer but also immediately interpretable in its biological context.*

17. The integration of the three omics layers (transcriptome, proteome, metabolome) appears somewhat artificial. Please elaborate on how their functional relationships are captured or modeled.

*ANSWER: We have expanded the Methods and Results sections to clarify how the multiome integration is performed and how functional relationships across transcripts, proteins, and metabolites are captured within the kernel-DMD framework.*

*In the revised manuscript, we explain that integration is not performed by simple concatenation alone. Instead, functional relationships across the three omic layers are modeled through:*

- *The nonlinear kernel, which embeds interactions among all features—irrespective of omics type—into a shared reproducing-kernel Hilbert space. This ensures that transcripts, proteins, and metabolites can influence one another through nonlinear pairwise similarities.*
- *The inclusion of a graph Laplacian term, which incorporates prior biological knowledge from STRING (PPI) and KEGG (protein–metabolite relationships). This ensures that cross-omic dependencies (e.g., transcript → enzyme → metabolite) influence the learned dynamics.*
- *The Koopman operator, which captures how multiome states evolve jointly over time. The resulting eigenmodes contain mixed multiome features because the operator models their coupled dynamics rather than treating each omics dataset independently.*
- *The multi-objective function, which forces all omics layers to contribute to predicting physiologically relevant outputs (malate, CO₂ exchange, PEPC, PCK1, etc.). This explicitly couples the layers through shared dynamical objectives, not statistical correlation.*
- *Consensus mode analysis, which demonstrates biologically coherent cross-omic groupings (e.g., glycolytic transcripts, TCA-cycle metabolites, and regulatory proteins clustering together in CAM eigenmodes).*

*These clarifications were added to the manuscript, and additional explanatory text has been incorporated to help the reader understand how the functional relationships across omics layers are encoded dynamically in the model.*

18. The concept of “control” in the system is not clearly defined. What does "control" biologically represent? Are there experimental validations for the model predictions, or are the results purely theoretical? How should readers interpret the reported "predictive accuracy"?

*ANSWER: In our study, control refers to data-driven perturbations applied to the internal state of the multiomic system that simulate how altering specific molecular features (genes, proteins, metabolites) would shift the system toward CAM-like dynamics.*

*More concretely:*

*The control matrix* *B derived from the nonlinear component of the model identifies directions in feature space that—if modulated—would cause the system to follow a different dynamical trajectory.*

*Biologically, this corresponds to hypothetical interventions, such as:*

- *up/downregulation of certain transcripts,*
- *increasing or decreasing enzyme activities,*
- *shifting metabolite pools,*
- *or targeted genetic modifications.*

*Thus, control does not refer to direct experimental manipulation performed in this study but to theoretical, model-driven perturbations that identify potential molecular levers enabling a transition from C3+CAM to CAM behavior.*

*We have added explicit text in both the Results and Discussion clarifying this.*

*The identified control features (e.g., PFK3, GAPN, FBA2, PEPC, PPCK1) are fully consistent with experimentally known regulators of C3↔CAM transitions reported in the literature (2-6), and the model confirms independent genomic findings from our prior work on Clusia species (7).*

*We have clarified in the revised text that experimental validation is a future step, and the purpose of the present work is computational hypothesis generation.*

*Regarding the term “predictive accuracy”, this refers specifically to: how well the controlled C. major system reproduces the experimentally observed outputs of C. rosea, such as malate accumulation, gas-exchange patterns, and abundances of key CAM enzymes. Thus, the R² or Pearson correlation coefficients quantify: The degree to which the predicted output trajectories after control match the real biological trajectories measured in the CAM species. They do not imply experimental validation of control inputs but measure the internal consistency and feasibility of the model’s predicted phenocopy.*

*We have now added explicit text stating this.*

1.	Baddoo PJ, Herrmann B, McKeon BJ, Brunton SL. Kernel learning for robust dynamic mode decomposition: linear and nonlinear disambiguation optimization. Proceedings of the Royal Society A: Mathematical, Physical and Engineering Sciences. 2022;478(2260):20210830.

2.	Nose A, Miyata A, Kobayashi K, Wasano K. Temperature and pH responses of phosphofructokinase from three CAM plants, Ananas comosus, Kalanchoë pinnata, and K. daigremontiana. In: Garab G, editor. Photosynthesis: Mechanisms and Effects: Volume I–V: Proceedings of the XIth International Congress on Photosynthesis, Budapest, Hungary, August 17–22, 1998. Dordrecht: Springer Netherlands; 1998. p. 3599-602.

3.	Ostrem JA, Vernon DM, Bohnert HJ. Increased expression of a gene coding for NAD:glyceraldehyde-3-phosphate dehydrogenase during the transition from C3 photosynthesis to crassulacean acid metabolism in Mesembryanthemum crystallinum. J Biol Chem. 1990;265(6):3497-502.

4.	Guan Q, Kong W, Zhu D, Zhu W, Dufresne C, Tian J, et al. Comparative proteomics of Mesembryanthemum crystallinum guard cells and mesophyll cells in transition from C3 to CAM. Journal of proteomics. 2021;231:104019.

5.	Guan Q, Kong W, Tan B, Zhu W, Akter T, Li J, et al. Multiomics unravels potential molecular switches in the C(3) to CAM transition of Mesembryanthemum crystallinum. J Proteomics. 2024;299:105145.

6.	Lüttge U. CO2‐concentrating: consequences in crassulacean acid metabolism. Journal of Experimental Botany. 2002;53(378):2131-42.

7.	Hannes M. Kramml JBH, Clara Priemer, Zoe Wessely, Florian Schindler, Andreas Berger, Maximilian Kellner, Stefan Plott, Tamara Schmidt, Peter Kerpan, Leila Afjehi-Sadat, Palak Chaturvedi, Arindam Ghatak, Martin Brenner, Iro Pierides, Lena Fragner, Eva M. Temsch, Fabio Trevisan, Menriti Ibrahim, Felix Fromwald, Anke Bellaire, Oleg Simakov, Werner Huber, Ulrich Lüttge, Ovidiu Paun, Susann Wicke, Hanna Weiss-Schneeweiss, Gert Bachmann and Wolfram Weckwerth. Clusia genomes shed light on the evolution and diversity of CAM physiotypes In: Department of Functional and Evolutionary Ecology UoV, editor. Vienna, Austria(in review).
